# Studying Alcohol Elimination Using the Alcohol Clamp Method

**Published:** 2006

**Authors:** Vijay A. Ramchandani, Sean O’Connor

**Affiliations:** Vijay A. Ramchandani, Ph.D., is a staff scientist and acting chief of the Unit on Human Physiology and Pharmacokinetics in the Laboratory of Clinical and Translational Studies, Division of Intramural Clinical and Biological Research, National Institute on Alcohol Abuse and Alcoholism, Bethesda, Maryland. Sean O’Connor, M.D., is a professor of Psychiatry at the Indiana University School of Medicine and a scientific director for Human Studies at the Indiana Alcohol Research Center and director of the Substance Abuse Treatment Section at the Richard L. Roudebush Veterans Affairs Medical Center, Indianapolis, Indiana

**Keywords:** alcohol absorption, ethanol metabolism, alcohol clamp method, alcohol elimination, alcohol elimination rate (AER), breath alcohol concentration (BrAC), intravenous infusion, pharmacokinetics, pharmacodynamics, Michaelis-Menten kinetics, alcohol dehydrogenase (ADH), acetaldehyde dehydrogenase (ALDH), genetic polymorphisms, liver, food intake

## Abstract

Researchers studying alcohol absorption and metabolism in humans have been aided by the alcohol clamp method, in which alcohol is administered intravenously, allowing study participants to achieve and maintain a target breath alcohol concentration (BrAC) for an extended period of time. This tool minimizes the variability in BrACs that occurs after alcohol consumption by administering alcohol at a dose and rate that is computed for each person individually. The alcohol clamp can be used to evaluate several influences on alcohol elimination, including gender, ethnicity, genetic variations in alcohol-metabolizing enzymes, and food consumption.

Multiple factors can influence breath alcohol concentration (BrAC)^1^ after alcohol use. To minimize the variability in BrACs that occurs in human research study participants after ingestion of alcohol, researchers developed an alcohol “clamping” method in which alcohol is administered intravenously to achieve and maintain a prescribed target BrAC. Individuals vary as much as three- to four-fold in the absorption, distribution, and metabolism of even a standardized oral dose of alcohol (Friel et al. 1995; Li et al. 1998). Many factors contribute to this variability, including those that can be controlled during an experiment such as the rate of input, type, concentration, and volume of alcoholic beverage consumed, and food intake (Dubowski 1985; Sedman et al. 1976). Other factors that are less controllable include first-pass metabolism of alcohol by the gut and liver prior to distribution into the bloodstream, anatomic and physiological variation in stomach (i.e., gastric) emptying, liver volume and blood flow, genetics, ethnicity, gender, age, and drinking history (Marshall et al. 1983; Li et al. 1998; Pikaar et al. 1988).

The alcohol clamp was developed to allow researchers to maintain a target BrAC in study participants despite these factors. With this research method, alcohol is intravenously administered (i.e., infused) for a prolonged, predetermined period of time (O’Connor et al. 1998; Ramchandani et al. 2006). Figure 1*A* shows a typical alcohol clamp, in which a target BrAC of 60 mg% (60 mg of ethanol in a 10th of a liter of fluid; 0.06 percent in terms related to automobile driving standards) is achieved in 10 minutes following the start of the infusion and then maintained at that level for 170 minutes. The desired BrAC exposure is achieved by administering alcohol according to a specific dosage and rate (i.e., infusion rate profile) (Figure 1*B*) that is pre-computed for each individual by estimating physiological parameters that include his or her specific alcohol elimination rate (AER). Researchers make this estimate using a computer model that describes what happens to a chemical in the body (i.e., a physiologically based pharmacokinetic [PBPK] model) for alcohol (Ramchandani et al. 1999; Plawecki et al. 2004). The model’s parameters are estimated for each subject, based on the individual’s age, height, weight, and gender. Using these estimates, a PBPK model–based algorithm computes the infusion rate profile for any desired BrAC exposure to be achieved during the experiment (for details of algorithm, please see Ramchandani et al. 1999 and O’Connor et al. 2000). The infusion profile, when administered to the study participant, yields the desired time course of BrAC. Ensuring that the target BrAC is accurately maintained requires minor real-time adjustments to the infusion rate profile. Such adjustments are based on serial breath alcohol measurements and are necessary in order to obtain BrACs that remain within 5 mg% of target levels (O’Connor et al. 2000) for intervals exceeding an hour.

## Overcoming Variation in Alcohol Absorption

Alcohol absorption is particularly influenced by the environmental sources of the variation across individuals outlined above (i.e., by rate, type, concentration, and volume of alcoholic beverage consumed and by food intake). As a result, assessment of the metabolic reactions responsible for alcohol elimination (i.e., elimination kinetics) of orally administered alcohol is confounded by the variability in alcohol absorption, especially because alcohol elimination rates nearly are independent of systemic alcohol concentration and follow Michaelis-Menten kinetics.^2^ Intravenous administration of alcohol circumvents absorption kinetics and provides a more direct assessment of alcohol metabolism in a steady state that cannot be achieved by oral administration. The alcohol clamp results in very similar breath alcohol exposures in every subject, which also facilitates the examination of the biochemical and physiological effects of alcohol (i.e., pharmacodynamics of alcohol) and their genetic and environmental determinants (Morzorati et al. 2002; Blekher et al. 2002; Ramchandani et al. 2002).

## Maintaining Steady State

The assessment of alcohol metabolic rates from the alcohol clamp experiment is based on steady-state principles. Steady state is defined for alcohol kinetics as the state where the rate of alcohol input (i.e., the infusion rate of alcohol) is equal to the rate of alcohol elimination from the body. Thus, during the latter part of an alcohol clamp, when the BrAC and the infusion rate both remain at steady state, the infusion rate of alcohol becomes a direct measure of the AER in grams per hour. The infusion rate of alcohol is the product of the concentration of alcohol in the solution being infused (i.e., infusate) and the current infusion pump rate.

Other methods of estimating the AER require assumptions about values of the subject’s Michaelis-Menten kinetics and volume of body fluid into which alcohol is distributed. The direct measurement of the AER during the clamped steady state does not require any such assumptions. Thus, the AER can be used to evaluate various determinants of alcohol metabolism, including gender, age, ethnicity, and genetic variations (i.e., polymorphisms) of the alcohol-metabolizing enzymes alcohol dehydrogenase (ADH) and aldehyde dehydrogenase (ALDH). The AER also can be used to examine the role of many other factors, such as recent food intake, lean body mass (LBM), liver blood flow, and menopausal changes in physiology, that contribute to variability in alcohol metabolism.

## Applications of the Alcohol Clamp

Based on the reasoning outlined above, the alcohol clamp method can be used to evaluate several intrinsic and extrinsic influences on alcohol elimination.

### Evaluation of Intrinsic Influences on Alcohol Elimination

#### Gender and Ethnicity

One of the first studies using the alcohol clamp to assess AER examined gender and ethnic differences in alcohol metabolism and the role of liver volume and LBM in explaining these differences (Kwo et al. 1998; Li et al. 2000; Sato et al. 2001; Ramchandani et al. 2001*a*). Eighty-five healthy, nonsmoking male and female social drinkers, stratified by ethnicity as Caucasian, African American, or Asian, underwent the alcohol clamp for measurement of AER at a target steady-state BrAC of 50 mg%. Each participant also underwent abdominal computed tomography scans for measurement of liver volume. The results indicated that AER (g/hr) was significantly higher in male than in female subjects, across ethnic groups. However, AER normalized per unit LBM (g/hr/kg) was significantly higher for female subjects, across ethnic groups. Consistent with this finding was the observation that female subjects had significantly higher liver volumes per unit LBM, compared with male subjects, across ethnic groups. This higher liver volume per unit LBM probably provides a relatively greater alcohol-metabolizing capacity per unit of LBM. The AER normalized per unit liver volume (g/hr/L) showed no significant gender differences in these healthy subjects. Currently, studies are underway to further understand these gender differences, as well as to evaluate the influence of other factors, including age and gender hormones, on alcohol metabolism.

#### Genetic Variations in Alcohol-MetabolizingEnzymes

The alcohol clamp also has been used to study genetic determinants of alcohol metabolism, including polymorphisms of the *ADH1B*, *ADH1C*, and *ALDH2* genes, which encode variants of the ADH enzyme. Neumark and colleagues (2004) examined the influence of the ADH1B*2 polymorphism on AERs in healthy young Israeli Jewish male subjects (Neumark et al. 2004; Ramchandani et al. 2006). Given the reported association of the ADH1B*2 variation (i.e., allele) in Jews with reduced alcohol consumption (Neumark et al. 1998; Shea et al. 2001; Carr et al. 2002) and a lowered risk of alcohol dependence (Hasin et al. 2002), as well as the high (19 to 32 percent) incidence of this allele variant in Jewish populations, the authors hypothesized that the presence of the ADH1B*2 allele would be correlated with a greater AER in young (19- to 33-year-old) healthy Jewish male subjects undertaking a 50-mg% alcohol clamp. Of 109 participants in the study, 32 percent carried the ADH1B*2 allele, including 4.6 percent who had two identical alleles (i.e., homozygotes). A significantly higher mean AER was observed among participants who carried the ADH1B*2 allele compared with ADH1B*1 homozygotes. The mean AER increased consistently (i.e., monotonically) with increases in the number of copies of the ADH1B*2 allele. Using a statistical procedure to determine the functional relationship between two or more variables (i.e., regression analysis), the ADH1B*2 allele alone explained 8.5 percent of the AER variance. The authors concluded that the use of the alcohol clamp enhanced the accuracy of AER estimates, revealing a genetic association that previously was undetectable. The demonstration of the genetic influence on AER encourages further examination of relative contributions of genes and environmental factors to alcohol metabolism and its effects and ultimately alcohol consumption in these populations.

Recent studies reported by Hesselbrock and colleagues (2005) also have used the alcohol clamp to examine the influence of ALDH2 genetic polymorphisms on AERs and the associated time-course of the concentration of the alcohol metabolism byproduct acetaldehyde in the blood of healthy Japanese male and female subjects. The authors also examined the influence of gender on the relationship between genetic makeup (i.e., genotype) and AER. The results confirmed previously reported gender differences in AER, in that male subjects had higher AERs than female subjects, although this difference disappeared after controlling for LBM. Male, but not female, subjects with two different alleles (i.e., heterozygous) for the *ALDH2* gene (ALDH2*1/*2) showed significantly lower AERs than those who were homozygous for the gene (ALDH2*1/*1). This suggests that the effect of the *ALDH2* polymorphism is apparent only in male subjects. Unlike previous reports, these results suggested no significant difference in LBM-normalized AER among *ADH1B* genotypes for either male or female subjects. Of note is the observation that although the BrAC remained clamped at a constant level of 50 mg%, the blood acetaldehyde concentration appeared to decrease over time in individuals with the less active *ALDH2* genotype. The reason for this decrease in acetaldehyde is unclear and suggests that other factors may play a role in acetaldehyde elimination in these individuals.

Given the central role of ADH and ALDH in the metabolism of alcohol and acetaldehyde, and the prevalence of genetic polymorphisms with physiological relevance for both *ADH1B* and *ALDH2* gene locations (i.e., loci), genotypic influences on AER can help explain important individual differences in the systemic levels of alcohol and acetaldehyde after alcohol consumption. More importantly, the AER should be evaluated as a genetically influenced marker (i.e., endophenotype) of more complex phenotypes related to alcohol use and misuse (Gottesman and Gould 2003; Hines et al. 2005). Little research has evaluated these relationships (Grant et al. 1999; Whitfield et al. 2001). Further studies are important for improving our understanding of how these heritable metabolic capacities contribute to the risk for development of alcohol dependence and possibly alcohol-related types of organ damage.

## Evaluation of Extrinsic Influences on Alcohol Elimination

The influence of environmental factors, such as food intake, on alcohol metabolism also has been examined using the clamp. Previous studies have reported that food intake decreases and delays systemic alcohol levels. However, most of these studies were conducted using oral alcohol administration and therefore were unable to distinguish between the influence of food on the absorption of alcohol or how much of the administered alcohol dose reaches the systemic circulation (i.e., bioavailability) and the influence on the metabolism of alcohol. By using intravenous infusions of alcohol, the alcohol clamp can provide a direct examination of the influence of food on AERs without the confounding effect of variability in alcohol absorption. Two studies (both described in Ramchandani et al. 2001*b*) have been conducted to evaluate the effect of food and food composition on AER in healthy men and women using the alcohol clamp. In the first of two studies, 10 male and 10 female nonsmoking, social drinkers underwent two alcohol clamp sessions at 50 mg% for measurement of their AER, one session following an overnight fast and the other following an approximately 530-calorie meal. Results showed a 25 to 30 percent *increase* in AER following the meal compared with the fasted state. In the second study, each of eight healthy nonsmoking social drinkers underwent four clamping sessions at 50 mg% for estimation of changes in AER under different food conditions. In each session, the alcohol clamp was preceded by an overnight fast, then by a continued fast or a high-fat meal, a high-carbohydrate meal, or a high-protein meal in randomized order. Comparison of AER estimates among the fed sessions showed that AERs following any meal studied consistently were about 45 percent higher than the AER from the fasted session but with no differential effect of meal composition on the AER. Probable mechanisms for the food-induced increase in alcohol elimination include increases in liver blood flow and increased ADH activity, perhaps through perturbation of the ratio of the reduced form of the coenzyme NAD (i.e., NADH) to the oxidized form of NAD (i.e., NAD^+^)^3^ in the liver. However, further studies are needed to understand the mechanisms underlying this food-induced change in AER.

## Perspective

Alcohol clamping has been performed quite safely in more than 1,000 study participants in laboratories across the world. The method requires insertion of an intravenous catheter into the area on the arm in front of the elbow and real-time management of both intravenous infusion pumps and BrAC meters. Knowledge of the precise concentration of beverage alcohol (i.e., ethanol) in the infusate, typically 6.0 percent by volume, is essential for computation of the AER or, in other studies, quantifying alcohol metabolism. Although faster methods are being developed, the clamping approach to assessment of AER depends on achieving a steady-state interval lasting approximately 45 minutes, and this interval cannot begin until distribution of alcohol equilibrates throughout the body. Thus, such experiments typically last 2.5 hours and fluid loading will require one or more bathroom breaks during the experiment. Achieving a steady state also is enhanced by the training and experience of the person performing the infusion and by the use of a PBPK model for pre-computation of a nominal infusion profile for each subject. The authors invite any investigators interested in using the method to contact either of us for help in shortening the learning curve.

We believe the alcohol clamp methodology provides a precise, accurate, reliable, and unique platform to study the metabolism of alcohol and to help obtain a better understanding of the sources of variance in the pharmacokinetics of alcohol. Because the pharmacokinetics of alcohol determine both systemic and brain exposure to alcohol, the alcohol clamp provides a useful tool in studies of determinants of the risk of alcohol dependence and alcohol-related problems.

## Figures and Tables

**Figure:**
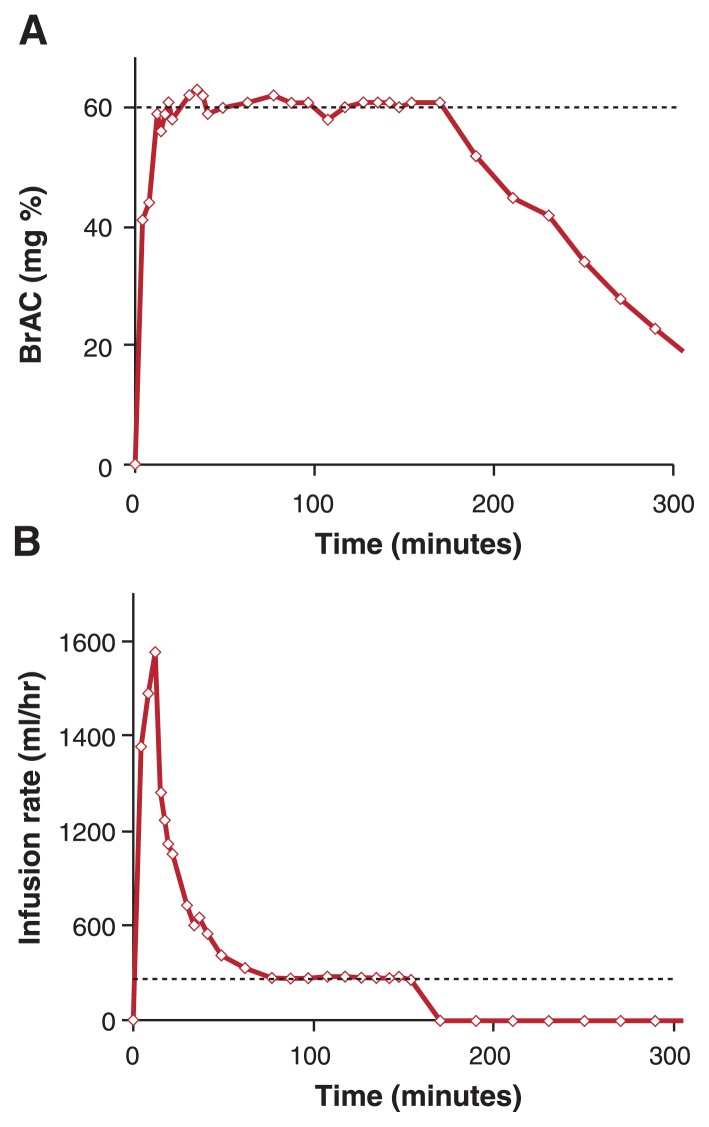
**A)** Typical alcohol clamp. Alcohol is administered intravenously (i.e., infused) to achieve and maintain a prescribed target breath alcohol concentration (BrAC). In this example, the target BrAC of 60 mg% is achieved in 10 minutes following the start of the infusion and maintained at that level for 170 minutes. **B)** Infusion rate profile (ml/hr of 6% vol/vol ethanol) individually estimated for each study participant to achieve the target BrAC exposure. The steady-state infusion rate (indicated by dashed line) is a direct measure of the alcohol elimination rate.

## Footnotes

1 Breath alcohol concentration is another way of quantifying alcohol levels in the body and is approximately the same as blood alcohol concentration after a given alcohol dose.

2 Michaelis-Menten kinetics is a model for describing the rates of biochemical reactions that are catalyzed by enzymes (i.e., enzyme kinetics). It is named for Leonor Michaelis and Maud Menten.

3 Cells produce NAD from niacin and use it to transport electrons in redox reactions, in which atoms either gain (reduction) or lose (oxidation) electrons. During this process NAD gains a pair of electrons and a proton and thus is reduced to NADH, releasing one proton (H^+^).
